# New Immunoinformatics Tools for Swine: Designing Epitope-Driven Vaccines, Predicting Vaccine Efficacy, and Making Vaccines on Demand

**DOI:** 10.3389/fimmu.2020.563362

**Published:** 2020-10-05

**Authors:** Lenny Moise, Andres H. Gutiérrez, Sundos Khan, Swan Tan, Matt Ardito, William D. Martin, Anne S. De Groot

**Affiliations:** ^1^EpiVax, Inc., Providence, RI, United States; ^2^Center for Vaccines and Immunology, University of Georgia, Athens, GA, United States

**Keywords:** vaccine, swine, immunoinformatics, T cell, epitope, SLA, infectious disease, immunity

## Abstract

Novel computational tools for swine vaccine development can expand the range of immunization approaches available to prevent economically devastating swine diseases and spillover events between pigs and humans. PigMatrix and EpiCC are two new tools for swine T cell epitope identification and vaccine efficacy analysis that have been integrated into an existing computational vaccine design platform named iVAX. The iVAX platform is already in use for the development of human vaccines, thus integration of these tools into iVAX improves and expands the utility of the platform overall by making previously validated immunoinformatics tools, developed for humans, available for use in the design and analysis of swine vaccines. PigMatrix predicts T cell epitopes for a broad array of class I and class II swine leukocyte antigen (SLA) using matrices that enable the scoring of sequences for likelihood of binding to SLA. PigMatrix facilitates the prospective selection of T cell epitopes from the sequences of swine pathogens for vaccines and permits the comparison of those predicted epitopes with “self” (the swine proteome) and with sequences from other strains. Use of PigMatrix with additional tools in the iVAX toolkit also enables the computational design of vaccines *in silico*, for testing *in vivo*. EpiCC uses PigMatrix to analyze existing or proposed vaccines for their potential to protect, based on a comparison between T cell epitopes in the vaccine and circulating strains of the same pathogen. Performing an analysis of T cell epitope relatedness analysis using EpiCC may facilitate vaccine selection when a novel strain emerges in a herd and also permits analysis of evolutionary drift as a means of immune escape. This review of novel computational immunology tools for swine describes the application of PigMatrix and EpiCC in case studies, such as the design of cross-conserved T cell epitopes for swine influenza vaccine or for African Swine Fever. We also describe the application of EpiCC for determination of the best vaccine strains to use against circulating viral variants of swine influenza, swine rotavirus, and porcine circovirus type 2. The availability of these computational tools accelerates infectious disease research for swine and enable swine vaccine developers to strategically advance their vaccines to market.

## Introduction

Pigs are an important component of the agricultural economy worldwide and are an important contributor to protein intake for populations living in developed and developing world economies. Due to the concentration of pigs in industrial farming operations and concern about the overuse of antibiotics for food animals, the control and prevention of infectious diseases in swine has become an important topic that is not only relevant to animal health and wellbeing but also to global food security and economic stability. Vaccine development for swine is likely to be facilitated by the emergence of computational tools for vaccine design. These same tools may also contribute to research on the spread of swine pathogens within herds and across geographical borders. For example, influenza is more diverse in swine populations than in humans. Spillover of influenza strains from pigs to humans was observed in 2009, and efforts to predict the next such event may be improved by comparisons of circulating strains in different species, a process that can be enabled by computational tools. Such tools may also contribute to the development of novel vaccines for important pathogens of swine for which effective vaccines are not yet available, such as African Swine Fever Virus (ASFV), a pathogen that is affecting swine populations in Asia and Europe (1).

Veterinary vaccines are one of the more cost-effective means of controlling, eradicating diseases and protecting herd health. Nevertheless, culling infected animals and strict containment are, in many instances, the only method available to limit the spread of disease during outbreaks (2). In order to move away from culling and quarantining infected animals, new types of vaccines and new vaccine methodologies that reduce the susceptibility of swine to infections bear serious consideration. Given the emergence of new strains of influenza and diseases that become endemic in new locations such as ASF in swine populations, and ethical considerations related to the culling of animals in industrial farming operations, there is a critical need for tools that can enable novel vaccine design, accelerate vaccine design, and assess the efficacy of vaccines against circulating strains, *in silico*.

Most veterinary vaccines are developed using standard methods, such as inactivating the pathogen using chemical or physical methods and then injecting killed organism directly into the animals (a process that can be called “shake and bake”). Alternatively, molecular tools are used to selectively modify a pathogen so as to limit virulence, resulting in an attenuated version that can be used as a vaccine. These vaccine approaches do not adequately address strain variation, which is a significant problem for the development of swine vaccines, as many of the pathogens affecting swine are highly variable. Additionally, viral pathogens have been shown to modify T cell epitopes to evade host immune response (immune escape) and more recently, selected epitope sequences of pathogens have been shown to resemble epitopes found in their hosts (immune camouflage) (3). Research in the field of human immunology has contributed to the development of tools that permit the evaluation of pathogen variation and immune camouflage. Although no examples of immune camouflage have been demonstrated in pigs, evolution of pathogens in pigs and the close resemblance of human and swine immune systems, including the Th1/Th2/Th17/Treg paradigm, suggests immune camouflage may occur in pigs like in humans. The availability of tools that discover pathogen epitopes that resemble their host sequences may lead to improvement in the process of antigen selection and enabling researchers to improve the efficacy of vaccines for swine.

Computational tools for vaccine design usually start with T cell epitope prediction due to the important role of T cell epitopes in cell-mediated immunity (CMI). T cell epitope mapping algorithms enable the analysis of complete proteomes of any size to identify vaccine candidates for experimental validation. Despite the demonstrated utility of computational vaccinology in human vaccine development (4), computational tools for vaccine design are very limited for non-human species. This is mainly due to the limitations on available experimental data that is required to develop prediction models. However, methods for extracting similarities between human and swine immune system orthologs exist and have been applied to develop new epitope prediction tools for swine (5), and this makes it possible to imagine further improvements in epitope-prediction models and further expansion of computational vaccinology tools. The fact that swine are both “patient” and “experimental model” facilitates the testing of hypotheses and will enable the development of at least as many applications of immunoinformatics tools as for humans and the acceleration of porcine immunology research.

Here, we review new immunoinformatics tools for swine developed by a team of scientists at EpiVax in partnership with researchers based in academic settings (University of Rhode Island, University of Georgia), that have been integrated into an existing toolkit for human vaccine design. The hybrid toolkit has been applied to design and evaluation of novel vaccines for influenza and African Swine fever, and to the analysis of vaccine for protective efficacy against circulating strains of influenza and porcine circovirus. We also discuss current challenges and future perspective in the field.

## The iVAX Toolkit

Computational vaccinology is a term that incorporates epitope mapping, antigen selection and vaccine construct design using computational tools. *In silico* tools are at the core but validation is used to improve the efficacy of prediction and to measure the impact on immune responses to pathogens. A wide range of tools have been developed in the past 20 years that dramatically accelerate the design of novel and next generation vaccines. In a recent publication, we have described the utility of iVAX for human vaccine design and analysis (4). Here we will focus on the integration of PigMatrix into a pre-existing toolkit, and describe applications of the combined tools to swine vaccines.

The iVAX toolkit has been in development since 1998. It is an interactive internet-based platform that integrates user input, immunoinformatics algorithms and several sequence databases, enabling users to rapidly identify and triage candidate antigens, select immunogenic T cell epitopes, eliminate potential regulatory T cell epitopes, and optimize antigens for immunogenicity and protection against disease. Detailed descriptions of the tools are published [see references (4, 6–8)]. While the tools were designed for humans, swapping out the tools used for epitope prediction from Human Leukocyte Antigen (HLA) to Swine Leukocyte Antigen (SLA) has enabled EpiVax vaccine developers to apply these advanced tools to infectious disease problems affecting swine.

### Overview of the iVAX Toolkit

**FIGURE 1:**
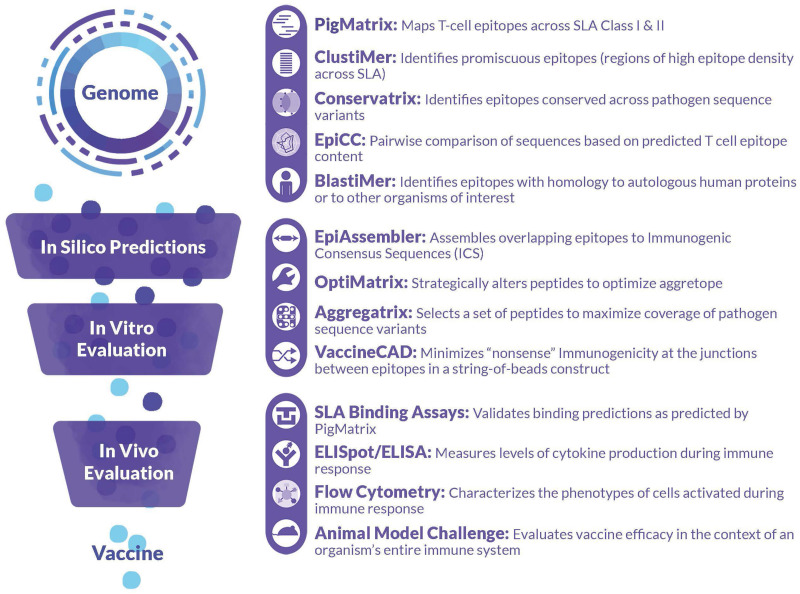
Integration of PigMatrix and EpiCC into the iVAX Toolkit. The iVAX toolkit is a comprehensive set of tools for *in silico* analysis and computational vaccine design for humans. PigMatrix analyzes protein sequences for Class I and Class II SLA-restricted T cell epitopes. EpiCC defines the relatedness of sequences based on their T cell epitope content. The integration of PigMatrix and EpiCC into the existing iVAX toolkit allows them to be applied for the development of accelerated and improved swine vaccines.

iVAX contains a compilation of tools that implement information derived from the T cell epitope mapping tool, EpiMatrix (9). This tool accepts sequence input for human, swine, and murine major hisotocompatability complex (MHC) class I and class II epitope prediction. The generated predictions can then be incorporated into further analysis using a variety of tools including the **Conservatrix, ClustiMer**, **EpiAssembler** (4) and **VaxCAD** algorithms (10). Conservatrix enables a search for sequences across variable pathogens, for example, swine influenza A, or Porcine Reproductive and Respiratory Syndrome Virus (PRRSV). ClustiMer finds regions of class II SLA-binding epitopes that cluster into a single longer sequence, and EpiAssembler is used for identifying epitopes that are conserved across several different strains of the same pathogen. Additional tools include **JanusMatrix**, a unique homology analysis tool that predicts the potential of a given peptide to contain epitopes exhibiting cross-reactivity between a pathogen and a host (such as swine) based on the conservation at the MHC-T cell receptor (TCR) interface. A list of the tools is provided in Figure 1 with a short description of their function.

### Immunogenicity Scale – Triaging Antigens

During the process of selecting candidate vaccine antigens, the overall immunogenic potential should be taken into consideration as it directly relates to the cytotoxic T cell (CTL) or T helper (Th) T cell epitope content. We have observed that the greater the concentration of HLA ligands and putative T cell epitopes that are contained in an antigen, the more likely it will induce an immune response.

**FIGURE 2:**
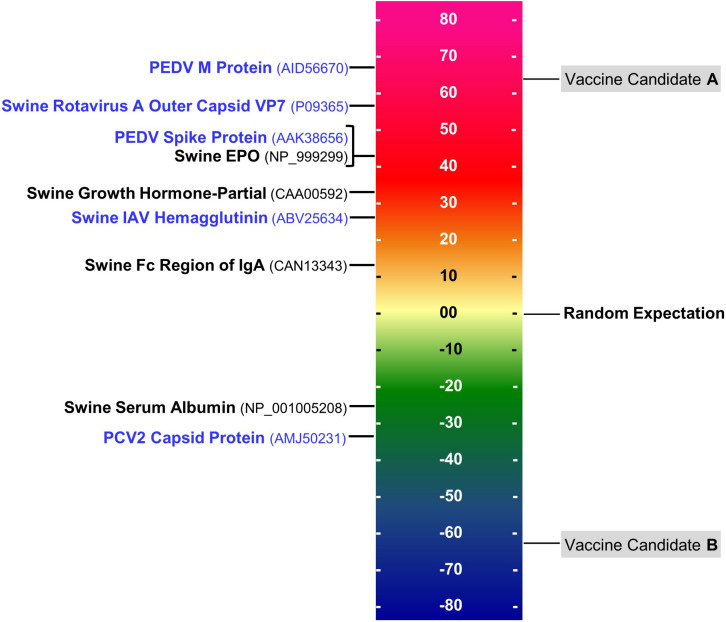
EpiMatrix immunogenicity scale. The immunogenicity scale shows swine pathogen antigens that have been reported to be immunogenic, and non-immunogenic antigens. Sequence accession numbers in GenBank are provided in the parentheses. The EpiMatrix immunogenicity scale is set to zero based on the average epitope content in a randomly generated protein sequence. Normalization of SLA scoring enables the ranking and direct comparison of candidate antigens; for example, candidate vaccine antigen A would be preferred over candidate vaccine antigen B for inclusion in a vaccine designed to elicit T helper immune response and to drive humoral response.

T cell epitope concentration can be expressed as an overall EpiMatrix score called the **EpiMatrix Protein Score**, which is the difference between the number of T cell epitopes predicted in a given protein and the number of T cell epitopes expected to be found in a random protein sequence, normalized for length (per 1,000 amino acids). The average number of T cell epitopes contained in 10,000 randomly generated protein sequences is set to zero, proteins considered to have a significant immunogenic potential score above 20 on the normalized scale, on which several swine pathogen antigens included for comparison, in Figure 2.

### Regional Immunogenicity

**FIGURE 3:**
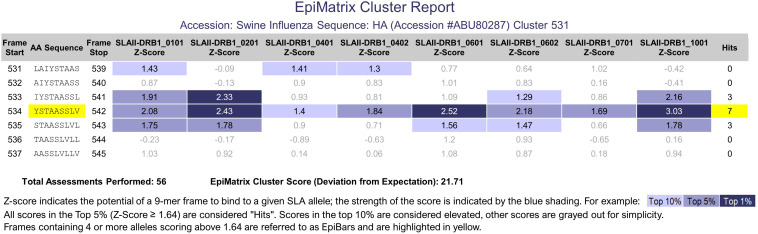
PigMatrix class II analysis. The cluster report of a swine influenza hemagglutinin sequence shows a 9-mer frame that contains three top 1% hits (strong binding likelihood) and four top 5% hits for SLA class II alleles. This feature is called an EpiBar and is characteristic of highly immunogenic epitopes.

While the normalized **EpiMatrix Protein Score** provides an approximation of the overall protein immunogenicity, regional immunogenicity also plays a role in the immunogenic potential. T cell epitopes tend to cluster in regions of protein sequences. **ClustiMer** was developed to identify regions with unusually high densities of putative T cell epitopes. For a given region, ClustiMer calculates a T cell epitope cluster score. Clusters with scores above 10 are considered potentially immunogenic. The length of T cell epitope clusters ranges from nine to approximately twenty-five residues and can contain from four to forty HLA binding motifs. T cell epitope clusters usually contain one or more 9-mer frame sequences predicted to bind to four or more HLA alleles. This epitope bar feature (EpiBar) is highlighted in the iVAX report. T cell epitope clusters can be highly immunogenic. An example is given of a Swine Influenza A Hemagglutinin epitope cluster (Figure 3). Human T cell epitope clusters that have a similar EpiBar have been defined for Tetanus toxin 825–850, GAD65 557–567 and are often used as controls for T cell assays (11). In our experience, these clusters are recognized in outbred populations of humans (12, 13); however similar epitopes have not yet been defined for swine.

### JanusMatrix and Self-Like T Cell Epitopes

Although T cells possessing anti-self TCRs were previously thought likely to be eliminated in the thymus, evidence emerged showing that anti-self immune response is also controlled by regulatory T cells recognizing the same antigens (14, 15). The phenotype of these regulatory T cells may be reinforced by repetitive re-exposure to their cognate self-antigens (16). Thus, immune response to new antigens is shaped by previous experience in the thymus and by exposure-driven reinforcement in the course of immune system maturation.

We observed that certain pathogens contain critical antigens with T cell epitopes that are highly conserved with self-antigens. This is true for humans and consequently deserves attention in swine. We hypothesized that pathogens use these epitopes as a means of “immune camouflage”; thus, these epitopes might be tolerated or actively tolerogenic upon vaccination (17). In retrospective studies, we determined that peptide epitopes that have identical TCR-facing residues and similar MHC binding anchors can be potentially tolerogenic and/or activate T cells that have a regulatory T cell phenotype or induce immunosuppressive responses (3). To identify these self-like epitopes, we developed the JanusMatrix tool. Using this tool, we are studying the impact of mutating these epitopes to enhance vaccine immunogenicity in humans (18) and anticipate that we will extend this work in collaborations that will evaluate the impact of self-like epitopes for swine.

**FIGURE 4:**
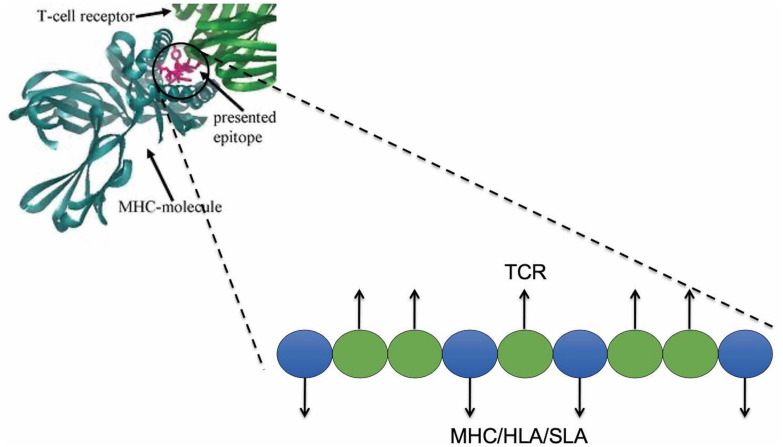
JanusMatrix and self-like epitopes. Predicted SLA ligand with identical TCR-facing residues with the swine proteome (presented in blue) and variant SLA-binding residues (presented in green) may stimulate cross-reactive tolerizing or Treg responses, if both bind to the same SLA allele.

For any given putative T cell 9-mer epitope, JanusMatrix analyzes residues in contact with the MHC molecule, and those in contact with the T cell receptor (TCR). Positions 1, 4, 6, and 9 are assumed to interact with MHC class II molecules and positions 2, 3, 5, 7, and 8 are assumed to interact with TCRs (Figure 4). For class I epitopes, the TCR-facing residues vary from allele to allele.

The JanusMatrix algorithm then searches a reference database for similar epitopes, considering both MHC- and TCR-facing residues. The reference database (to which pathogen epitopes are compared) can be human, swine, murine, or any other organism (including other pathogens from the same, or similar species). JanusMatrix finds reference epitopes with identical TCR-facing residues that are predicted to bind to the same MHC molecule despite amino acid differences. JanusMatrix calculates a Homology Score as the average depth of coverage within the reference database for the putative MHC binding epitopes identified in the input peptide. JanusMatrix Homology Scores above two are considered to be significant, indicating an elevated level of conservation between putative epitopes in the input peptide and epitopes in the reference database. Using this threshold, we identified epitopes that are more likely to be tolerated or actively regulatory (19). For a given EpiMatrix Score, a high JanusMatrix Homology Score suggests that T cells recognizing that epitope may exhibit a bias toward immune tolerance, which has been validated in retrospective and prospective studies (3) in the human context. More remains to be done to evaluate whether the same observation is true in swine.

## PigMatrix

In general, the development of models for prediction of T cell epitopes requires a large amount of experimental data for training and testing. A variety of approaches provide data that can be used to define peptide:MHC binding rules and enable binding predictions, all of which have been applied to identification of SLA ligands. High throughput methods that define MHC binding peptides include biochemical assays that measure peptide:MHC binding affinity or a proteomics approach that uses immunoprecipitation of solubilized peptide:MHC complexes from the cell surface followed by peptide elution and liquid chromatography/mass spectrometry (20–22). Additionally, in a low throughput manner, epitope-specific T cell lines are used to define binding anchor residues by assaying epitope variants at anchor positions for T cell stimulation as measured by cytokine or chemokine release (23). While such binding data are abundantly available for HLA, they are limited for MHC of other species. Only one online tool algorithm has been trained and evaluated for prediction of SLA class I alleles (24). Prediction tools have not been available for SLA class II alleles. To overcome this lack of binding data for SLA, PigMatrix leverages similarities between the secondary structure of HLA and SLA molecules and predefined HLA binding preferences to generate SLA epitope predictors based on the pocket profile method (5, 25).

The crystallographic structure of HLA molecules reveals that the peptide-binding groove contains a number of pockets and that polymorphic residues in the HLA sequence are often involved in forming these pockets (26). Consequently, the residues in the pocket define allele-specific binding preferences for particular amino acid side chains of the antigenic peptides (27). Thus, for each MHC molecule, the profile of a given binding pocket can be defined by its residues and binding preferences. Sturniolo et al. demonstrated that each “pocket profile” was nearly independent of other pockets in the HLA-DR binding groove (25). The authors also showed that an MHC molecule could be defined in terms of its individual pocket profiles as a quantitative matrix of binding preferences. Therefore, once a pocket profile is determined experimentally, it can be shared with other HLA-DR molecules that have identical pocket residues.

A number of pan-specific algorithms for T cell epitope prediction based on the pocket profile method have been developed, including TEPITOPE (25), TEPITOPEpan (28), and PickPocket (29). The predictive performance of these methods for novel HLA alleles depends on the similarity of pocket residues; performance decreases as similarity decreases (29). For HLA alleles with limited quantitative data, algorithms based on the pocket profile method have demonstrated better or comparable performance when compared to methods, such as artificial neural networks, that require a large amount of training data (28, 29). NetMHCpan, an artificial neural network-based algorithm, has been used for prediction of SLA class I-restricted peptides (24, 30).

PigMatrix (5) is the first algorithm that was designed for the prediction of SLA class II T cell epitopes. Using the Sturniolo et al. approach described above, PigMatrix matrices were created by integrating the binding preferences of the best-matched HLA pocket for each SLA pocket, using SLA or HLA crystal structures as a basis for pocket selection. PigMatrix achieved a favorable predictive performance, comparable to or better than PickPocket and NetMHCpan for SLA class I alleles (5). PigMatrix class II epitope predictions were validated prospectively (see section “Swine Influenza A Virus Vaccine” below). Overall, using the pocket profile method for SLA, and defined binding preferences from HLA, shows promise for developing T cell epitope prediction tools for pigs.

### Limitations of PigMatrix: Class I and II SLA Coverage

To effectively harness epitope immunoreactivity data, the identity of SLA alleles involved in peptide presentation to T cells is required. This information is needed to establish knowledge of the prevalence of allelic families on a population level, which is used in turn to ascribe immunological significance to epitope-specific T cell responses detected in infection and vaccine studies. Furthermore, knowledge of MHC allele sequences is required for T cell epitope prediction.

The diversity of SLA and the lack of information on SLA frequencies represent a significant challenge for the development of T cell epitope vaccines for swine (31). The problem of SLA coverage is illustrated by a small swine influenza vaccine immunogenicity study that was performed using PigMatrix-identified T cell epitopes, SLA alleles expressed by the pigs in the study cohort were different from those reported to be prevalent in the United States swine population. Information about SLA allele diversity in the United States swine population is critically important to develop a more comprehensive set of predictions that target the most prevalent SLA alleles. Once the prevalence and diversity of United States swine SLA are better understood, it may be possible to cluster SLA molecules into supertypes. The concept of supertypes has been applied to HLA for selection of few representative alleles from different clusters to cover a high percentage of the HLA diversity in the human population (32, 33). An epitope-based vaccine containing peptides predicted to bind SLA supertype alleles could induce immune responses in pigs expressing diverse alleles.

Fortunately, the importance of SLA diversity for vaccine development and studies to identify commonly expressed haplotypes has been recognized and new studies are expanding available information on prevalent SLA alleles in swine poulations (34). Currently, the Immune Polymorphism Database lists 90 SLA-1, 96 SLA-2, 41 SLA-3, and 99 DRB1 alleles. Continuing efforts to expand the identification of specific alleles are needed, as are studies that will determine allelic frequencies on a population level for prediction of T cell epitope binding for vaccine development and analysis of epitope-specific T cell responses in infection and vaccination.

SLA typing is commonly performed using sequence-specific primers in PCR (PCR-SSP) (35, 36). This is a labor-intensive approach that yields low resolution results at the allele group level; e.g., SLA-1^∗^08XX refers to a group of alleles that encode the SLA-1^∗^08 antigen or sequence homology to other SLA-1^∗^08 alleles. Improved resolution to four digits is needed to identify specific allele proteins (e.g., SLA-1^∗^0801, SLA-1^∗^0802). High-resolution and high-throughput methods have also been developed (37). Next generation sequencing is a widely used technology for HLA typing (38, 39) and has been used for SLA typing in a few studies (40, 41). A commercially available high-throughput method for high-resolution SLA-typing would improve the ability of researchers and producers to determine SLA diversity.

## Epitope Content Comparison (EpiCC)

Using PigMatrix, it is possible to identify potential T cell epitopes and rank proteins based on their immunogenic potential. In addition to immunogenicity, vaccines need to induce memory T cells that will recognize epitopes contained in circulating strains. In other words, the epitope content of a vaccine should be similar to that of the circulating strains to elicit broad immune recognition and protection.

To estimate the relationship between pathogen sequences based on their putative T cell epitope content and predict cross-protection potential, we developed the T cell Epitope Content Comparison tool (EpiCC) which facilitates sequence pairwise comparison based on epitope content rather than sequence identity (42). EpiCC assesses the relatedness of T cell epitopes contained in a protein sequence of one strain and those in another based on a comparison of the epitope sequences and their PigMatrix SLA binding score. T cell epitopes can be either shared (cross-conserved) between sequences, or unique to each strain. Thus, the EpiCC score for the comparison of two strains is based on the PigMatrix scores of shared and unique epitopes, which are defined using JanusMatrix. For a pair of protein sequences, the EpiCC score is high if the epitope content shared between both sequences is dense and similar. For comparison of a vaccine and outbreak strains, vaccine sequences that share more T cell epitope content with circulating strains have higher EpiCC scores.

EpiCC can be applied to estimate whether a given vaccine would protect against circulating or newly emerging strains of a pathogen. It can also potentially be used to assist in the selection of live or killed organism vaccine candidates by comparing one or multiple antigens and identifying the vaccine strain sequence that best represents the T cell epitope content of circulating strains and that may induce the broadest cross-reactive T cell response. See for example, the publication by Bandrick, M. et al., comparing monovalent and bivalent PCV2 vaccines to field strains (43). EpiCC also has applications for analysis of large-scale surveillance data to identify circulating or novel viruses distantly related to current vaccines for further experimental evaluation to determine potential risk of vaccine failure.

## Case Studies

### Vaccine Development Against Swine Pathogens Using the iVAX Toolkit

#### Swine Influenza A Virus Vaccine

Influenza A virus (IAV) is considered one of the most important infectious disease agents affecting North American swine (44). The majority of currently licensed swine IAV vaccines consist of whole inactivated viruses administered with adjuvants by intramuscular injection (45). This platform primarily induces systemic IgG antibody responses to the surface glycoproteins, mainly HA (45, 46). However, antibody-mediated immunity does not typically provide protection against divergent strains of IAV (46, 47). In contrast, CMI can be broadly cross-reactive to a variety of IAV subtypes (48, 49). Moreover, CMI contributes to virus clearance, reduces symptom severity, and virus shedding (50). A vaccine that can induce CMI and reduce morbidity could prevent anorexia and weight loss in swine, which cause significant economic loss to pork producers. Therefore, the identification of T cell epitopes conserved in diverse strains of IAV represents the first step toward the development of a potentially broadly protective vaccine.

Using PigMatrix and Conservatrix, the complete proteomes of representative IAV strains in a United States swine population were screened for class I and II T cell epitopes (31). EpiAssembler was used to construct immunogenic consensus sequences - peptides of 16–25 amino acid containing SLA-DRB1-restricted epitopes that were highly conserved in IAV strains, predicted to bind to multiple alleles, and enriched for immunogenicity. Using VaxCAD, 28 class I and 20 class II predicted epitope sequences were concatenated into two multi-epitope genes (one for SLA class I and one for class II epitopes). Cleavage promoting spacers or binding inhibiting “breaker” sequences were introduced where VaxCAD reordering did not eliminate junctional immunogenicity. Vaccine genes were synthesized and subcloned into vectors containing signals for proteasome or secretory pathway targeting.

The immunogenicity of the 48 predicted T cell epitopes was determined by measuring IFNγ recall responses using PBMCs from pigs immunized intramuscularly with the prototype DNA vaccine. Positive responses were observed upon restimulation with pooled peptides as well as eleven individual peptides. Recall responses to peptides were not observed in pigs immunized with a tetravalent inactivated commercial vaccine, despite containing similar internal antigens. This result suggested that the epitope-based DNA vaccine promoted more efficient processing and presentation of its own epitopes as compared to whole-protein-based vaccines.

In a vaccine challenge study, intradermal immunization with the epitope-based DNA vaccine followed by an intramuscular tetravalent inactivated vaccine boost was effective against H1N1 homosubtypic challenge. Pigs had reduced lung lesions and no detectable IAV antigen at necropsy. Moreover, IFNγ secreting cells, recognizing vaccine epitope-specific peptides and pH1N1 challenge virus were highest in PBMCs from pigs vaccinated using the prime-boost approach (51).

#### African Swine Fever Vaccine

African swine fever virus (ASFV) is the etiological agent of African swine fever (ASF), a highly contagious hemorrhagic disease of swine that affects domestic pigs and wild boars of all ages and breeds. Several clinical forms of ASF are presented in swine and include a hyper-acute or acute disease, a sub-acute disease and a chronic disease with mortality rates ranging from 100 to 3% depending on the virulence of the viral isolate, route of infection, and the host (52, 53). ASFV transmission to unexposed domestic pigs occurs by direct contact with an infected animal or the body fluids and carcasses of infected animals, or by indirect contact with contaminated materials or through the consumption of contaminated products (54). Wild pigs and soft ticks of the genus *Ornithodoros* are the natural reservoir for the ASF virus (55).

ASF poses a devastating threat to the global pig industry and has been spreading at an alarming rate in the past few years, affecting more than 55 countries in three different continents: Africa, Asia, and Europe (56). The introduction of ASF into these countries has dramatically impacted their socio-economics, pig production and status for international trade (57). Prevention, control, and eradication measures for ASF are mainly based on early detection and on the implementation of strict sanitary measures (58). However, successful control of ASF has proven to be challenging and the risk of introducing the virus into ASF-free countries is increasing. A vaccine against ASF is urgently needed to improve prevention and control strategies and mitigate major economic losses in endemic and non-endemic areas.

No licensed vaccine currently exists against ASF. The complexity of the virus and the large number of encoded proteins, with some involved in the modulation of host immune responses (59, 60), has made it challenging to identify immunogenic targets and hindered the development of an efficacious ASF vaccine. Another challenge is the genetic diversity of the ASFV and the limited knowledge of antigens involved in conferring cross-protection. Thus far, little to no cross-protection has been reported (61–63); however, pigs that survive ASFV infection generate protection against subsequent infections with a homologous ASFV (58). Several efforts have been made to develop an ASF vaccine with a current focus on the induction of both humoral and cellular immune responses due to their potential role in conferring ASF protection (64–67).

Using iVAX, we developed a T cell-directed ASF vaccine composed of swine MHC class I and class II epitopes conserved across 21 European, Asian and African isolates covering genotypes I, II, IX, and X. T cell epitopes identified by JanusMatrix as potentially regulatory (highly cross-conserved with the swine proteome) were excluded. Multi-epitope genes encoding class I and class II epitopes separately were each subcloned into plasmids to produce a DNA vaccine. The vaccine has undergone immunogenicity testing and is immunogenic (unpublished collaboration); further development is currently anticipated in collaboration with a commercial animal vaccine company.

### Applications of EpiCC

#### Swine Influenza A Virus Vaccine Analysis

For influenza and other viruses, sequence data and antibody cross-reactivity are commonly used to predict vaccine-induced protection (45, 46). However, previous efficacy studies demonstrated that even in the absence of cross-reactive antibodies, a commercial swine IAV vaccine was capable of inducing protection or partial protection (reduced lung lesions, reduced viral titers in lungs and/or nasal swabs) against heterologous challenge strains (46, 68–72).

**FIGURE 5:**
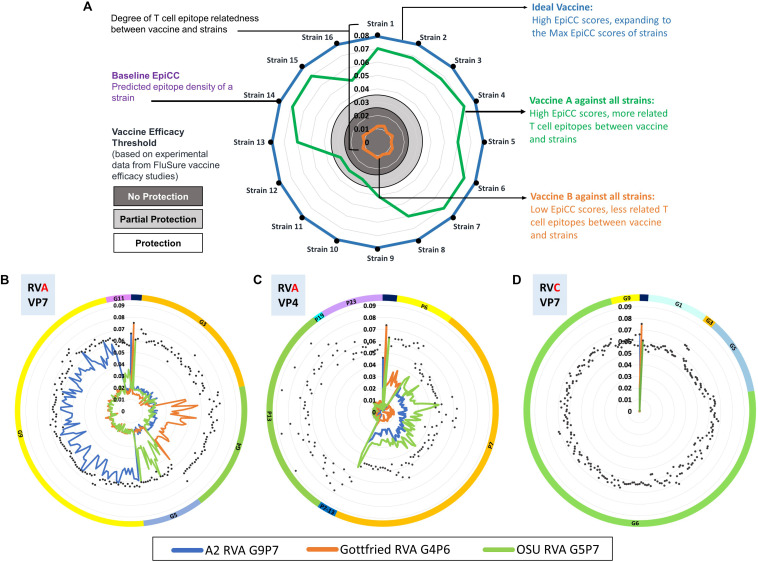
EpiCC radar plot. Radar plots are used to visualize the relationship between vaccine strains and circulating strains. In the top plot, Panel **(A)** for illustration purposes, 16 typical circulating swine influenza A virus strains are presented on the perimeter of the chart. The EpiCC scores of their HA antigens (hemagglutinin) are indicated by their distance from the center to the perimeter. Vaccine efficacy thresholds for protection (white area), partial protection (light gray area) and no protection (dark gray area) have been based on experimental data from efficacy studies of swine Influenza A virus vaccines (17). The blue line represents an example of an ideal vaccine strain that contains T cell epitopes fully matched to all the circulating strains. The green line represents an example of an influenza vaccine strain HA protein that contains T cell epitopes well matched to the majority of circulating strains. The orange line represents an example of a vaccine strain HA protein that contains T cell epitopes not well-matched to any of the circulating strains. This example is intended to illustrate how EpiCC provides guidance on vaccine selection but does not provide data on any specific influenza strains. An example of how EpiCC can be used is provided for a set of swine rotavirus vaccines and circulating rotavirus strains. Rotaviruses (RVs) are among the most common causes of acute diarrheal disease in humans and swine. Speciation of RVs is based on sequencing of the viral protein (VP) 6, the middle capsid protein. Rotavirus group A (RVA) is the most prevalent and pathogenic species of RV. The VP7 and VP4 proteins stimulate neutralizing antibodies and are used as a binary classification system for genotypes (G and P genotypes, respectively). Due to the binary classification system, we have performed an EpiCC analysis based on comparisons of the VP7 and VP4 components of each strain and their equivalent viral protein-specific vaccine components VP7 and VP4. In Panel **(B)**, we compare RVA strain VP7 proteins to the VP7 component of the vaccine, and in Panel **(B)** we compare RVA strain VP4 proteins to the VP4 component of the vaccine. The viruses are sorted by genotype (by G for Panel **(B)** and by P for Panel **C**); the classification is highlighted by the color of the outermost circle (orange for G3 and green for G4 and so on). Each of the three RVA strains in the ProSystems vaccine is represented with a different colored line: the blue line represents the A2 RVA strain which contains viral proteins derived from genotypes G9 and P7), the orange line the Gottfried RVA strain (which contains G4 and P6) and the green line the OSU RVA strain (G5 and P7). In Panel **(B)**, the EpiCC scores of the A2 vaccine strain (G9P7) are highest against strains that fall into the same genotype (G9) and low for all other genotypes. The EpiCC scores of the Gottfried strain (G4P6) are highest for strains that are in genotype G4 but low against other strains. This suggests that vaccine strains are more related to homologous field strains than to other strains. Therefore, the T cell epitope content of circulating swine rotavirus strains is highly genotype specific explaining why it is necessary to use genotype-specific RVA vaccines to protect against field strains. Panel **(D)** illustrates the expected finding that swine RVA vaccine strain VP7 has no conservation against circulating strains from rotavirus group C (RVC) VP7. If T cell epitopes are protective against swine rotavirus, a ‘universal’ RV vaccine would need to include T cell epitopes representing all of the genotypes.

To determine the potential role of T cell epitope-driven CMI in vaccine-induced protection in the absence of cross-reactive antibodies, an EpiCC analysis was performed to compare the T cell epitope content of HA sequences from swine IAV strains representing the major H1 clusters circulating in the North American swine population and those of H1 viruses in a commercial vaccine. Using experimental data from previous vaccine efficacy studies testing one of the H1 viruses in the commercial vaccine against different challenge viruses (46, 68–70, 72), a threshold level of T cell epitope relatedness associated with protection was identified. The published results provided supportive evidence that T cell epitopes that are conserved between vaccine sequences and circulating strains contributed to vaccine efficacy. We have provided a typical EpiCC analysis, using example influenza vaccines and strains, for illustration purposes, in Figure 5A.

For the initial influenza study, EpiCC analysis was restricted to HA sequences from 23 viruses representing diverse clusters of field strains, assuming limited T cell epitope variation of other antigens. However, the same approach is currently being applied to multiple antigens or to complete proteomes of influenza strains, and to hundreds of variant strains representing other pathogens such as PCV2. We anticipate that EpiCC may complement existing methods for vaccine selection in outbreak situations and could be used by animal vaccine companies for strain selection during vaccine development.

#### Swine Rotavirus Vaccine Analysis

We have also applied EpiCC to understand vaccine strain selection for swine rotavirus. Swine rotavirus serogroups A and C (RVA and RVC, respectively) are a significant cause of piglet morbidity and mortality across the world. The outer capsid of the RV particle is composed of the viral proteins VP7 and VP4, both of which are targets for neutralizing immunity and they also determine the G and P genotypes of RV strains (73). Cross-protection between RVA and RVC is non-existent while heterotypic immunity across different G and P genotypes remains limited (74). Given the large genetic diversity of RV genotypes, vaccination efforts have been limited. There is one currently available commercial vaccine that only contains three strains of RVA (75, 76). RV vaccine strains with high T cell epitope conservation with circulating strains may induce broader cross-protective immunity. Using EpiCC and PigMatrix, we investigated the presence of SLA class II putative T cell epitopes in the VP7 and VP4 of circulating porcine RVA and RVC strains and assessed the degree of their cross-conservation with the RVA strains in the ProSystems Rota vaccine (77). This data is shown in Figures 5B,C.

To perform this analysis, we first used PigMatrix to identify SLA class II-restricted T cell epitopes in a set of VP7 and VP4 proteins of RVA and RVC strains circulating in the United States as well as in the RVA strains Gottfried (G4P[6]), OSU (G5P[7]) and A2 G9P[7]) (76) that are used in the ProSystems Rota vaccine. We then performed an EpiCC analysis to assess the relationship between the T cell epitopes found in VP7 and VP4 of circulating RVA strains and the T cell epitope content of the RVA vaccine. The analysis demonstrated that T cell epitope cross-conservation between circulating strains and the RVA vaccine is genotype-specific and is limited to homologous strains as seen in (Figures 5B,C). In other words, T cell epitopes from the vaccine’s G9 genotype strain (called A2) are only conserved with field strains that belong to the G9 genotype, and this was also true for the G4 (Gottfried) and G5 (OSU) vaccine strains. There was very limited conservation between the T cell epitopes of the VP7 protein in the RVA vaccine with T cell epitopes found in non-homologous VP7 proteins in other genotypes of RVA (Panel B). This was also true when the VP4 protein is considered (Panel C). Thus, the existing RVA vaccine has genotype-specific T cell epitope content.

We then performed the same EpiCC analysis to assess the relationship between the VP7 of circulating RVC strains and RVA vaccine strains. The results again show that swine RVA VP7 T cell epitopes are serogroup specific and are not at all cross-conserved with the VP7 of RVC strains (Figure 5D). This study demonstrates that T cell epitopes found in circulating swine and vaccines are serogroup and genotype-specific, and may explain why vaccines to protect against swine rotavirus have to be multivalent.

#### Porcine Circovirus Type 2

Porcine circovirus type 2 (PCV2) is one of the top infectious agents in the porcine industry. Eight PCV2 genotypes have been described based on ORF2 phylogenetic analysis (78). Due to its remarkable evolutionary rate, further genetic variation of PCV2 is expected, limiting the usefulness of single vaccine strains. Currently, PCV2a, PCV2b, and PCV2d are considered to be clinically relevant causes of disease in swine populations, and PCV2d is currently the predominant genotype. However, most of the commercial vaccines available are based on the PCV2a genotype (79).

PCV2 vaccines were based on the 2a genotype because this was the first genotype that was discovered. Currently, eight genotypes of PCV2 viruses are known to circulate in swine populations, and further variation in PCV2 is expected. For these reasons, there is a need to determine how well existing and future vaccines cover field strains. We therefore used EpiCC to analyze the sequences of two major structural proteins, the replicase (encoded by ORF1) and the capsid (encoded by ORF2) from selected vaccines and compared the epitopes in the vaccines to those found in field strains. The two commercial vaccines that were analyzed in this study were based on PCV2a, PCV1-PCV2a chimeric virus (cPCV2a), an experimental PCV1-PCV2b chimeric virus (cPCV2b), and an experimental combination of cPCV2a and cPCV2b provided by the study’s co-authors at Zoetis.

The putative T cell epitope content of these vaccines was compared to that of 161 field strains representing PCV2 genotypes a-f using EpiCC (43). The analysis, performed using EpiCC and PigMatrix, demonstrated that the combination cPCV2a-cPCV2b vaccine had, on average, the highest EpiCC score against circulating strains. EpiCC scores of this vaccine were higher than those of the monovalent vaccines not only for PCV2a and PCV2b, but also PCV2d, which suggested that developing the combination vaccine would be preferable to developing a monovalent vaccine against the predominant circulating strain. EpiCC analysis suggested that the combination of cPCV2a and cPCV2b would confer the broadest cross-reactive cell-mediated immunity and protection against field strains (43).

## Conclusion

Recent developments in computation and genomics usher in new opportunities to address these unmet needs using immunoinformatic tools for accelerated design of safe and effective vaccines starting from sequence data. However, more research is needed. For example, further development of PigMatrix is necessary, to enable prediction for the broad range of SLA alleles that exist in global pig populations. Larger datasets of SLA-restricted peptides are required to further evaluate the PigMatrix approach and improve predictions. To generate quantitative binding data and test PigMatrix, binding assays for commonly expressed SLA molecules could be developed. Currently, these assays have been developed for a limited number of SLA class I and II alleles (24, 80–82). Binding assays provide valuable information to better define binding preferences and potentially develop predictions based on SLA specificities rather than pocket preferences. High-throughput binding assays using planar peptide microarrays have been applied to produce large amount of data (83). This technology could generate the data required to train and test SLA-specific models. One of the most significant interventions that would promote progress on new epitope-prediction models for additional SLA would be funding to carry out these studies.

Improvements to current methods of vaccine development are needed to protect swine from devastating pathogens and to stabilize the global food supply. Introduction of PigMatrix into the iVAX vaccine design platform has enabled demonstration of a heterologous prime-boost immunization strategy that protects against IAV and can be applied to other pathogens (51). Additionally, integrating PigMatrix into iVAX enables the comparison of related strains of highly variable pathogens to guide rational selection of candidate vaccine strains to advance to field trials and implementation. These novel computational tools are a valuable resource for countering pig-associated zoonotic disease to lower burden on pig production and human health.

In the context of epidemic outbreaks of infectious diseases, SLA-restricted epitopes can be identified and vaccines designed in under 48 h (84). Therefore, this computational “vaccines on demand” approach can be applied to other swine diseases of economic importance to accelerate vaccine development timelines by rapidly generating vaccine designs ready for production and testing. We note that requests for access to the tools for academic research can be directed to the University of Georgia technology transfer office, where two of the authors (ADG and LM) now have faculty appointments.

As illustrated here, vaccine design using the PigMatrix and the iVAX toolkit, may offer some advantages over standard approaches to developing vaccines for pathogens affecting the pork industry. PigMatrix and iVAX tools can be used to (i) accelerate vaccine design for new and emerging pathogens; (ii) identify highly conserved epitopes from the sequences of diverse strains that are able to drive cross-protective immune responses, reducing the need for developing a vaccine for each new strain of a pathogen; (iii) identify potential regulatory T cell epitopes; (iv) improve existing vaccines by engineering in more T cell epitopes or removing regulatory T cell epitopes; and (v) to predict the efficacy of existing vaccines against newer circulating strains of pathogens.

## Author Contributions

All authors listed have made a substantial, direct and intellectual contribution to the work, and approved it for publication.

## Conflict of Interest

AD and WM are senior officers and shareholders, and LM, AG, SK, and MA are employees of EpiVax, Inc., a privately owned biotechnology company located in Providence, RI. These authors acknowledge that there is a potential conflict of interest related to their relationship with EpiVax and attest that the work contained in this research report is free of any bias that might be associated with the commercial goals of the company. The iVAX toolkit is currently available for use by commercial developers by subscription or for specific projects under a fee-for-service arrangement. Academic researchers are invited to contact the authors at University of Georgia (UGA), or the technology transfer office at the UGA School of Veterinary Medicine, for access to the iVAX Toolkit for research purposes. The remaining author declares that the research was conducted in the absence of any commercial or financial relationships that could be construed as a potential conflict of interest.

## References

[1] Dixon LK Sun H Roberts H. African swine fever. *Antiviral Res.* (2019) 165:34–41. 10.1016/j.antiviral.2019.02.018 30836106

[2] Guinat C Vergne T Jurado-Diaz C Sánchez-Vizcaíno JM Dixon L Pfeiffer DU. Effectiveness and practicality of control strategies for African swine fever: what do we really know? *Vet Rec.* (2017) 180:97. 10.1136/vr.103992 27852963 PMC5293861

[3] Losikoff PT Mishra S Terry F Gutierrez A Ardito MT Fast L HCV epitope, homologous to multiple human protein sequences, induces a regulatory T cell response in infected patients. *J Hepatol.* (2015) 62:48–55. 10.1016/j.jhep.2014.08.026 25157982

[4] De Groot A Moise L Terry F Gutierrez A Hindocha P Richard G Better epitope discovery, precision immune engineering, and accelerated vaccine design using immunoinformatics tools. *Front Immunol.* (2020) 11:442. 10.3389/fimmu.2020.00442 32318055 PMC7154102

[5] Gutiérrez AH Martin WD Bailey-Kellogg C Terry F Moise L De Groot AS. Development and validation of an epitope prediction tool for swine (PigMatrix) based on the pocket profile method. *BMC Bioinformatics.* (2015) 16:290. 10.1186/s12859-015-0724-8 26370412 PMC4570239

[6] De Groot AS Jesdale B Martin W Saint Aubin C Sbai H Bosma A Mapping cross-clade HIV-1 vaccine epitopes using a bioinformatics approach. *Vaccine.* (2003) 21:4486–504. 10.1016/S0264-410X(03)00390-6 14505932

[7] De Groot AS Bishop EA Khan B Lally M Marcon L Franco J Engineering immunogenic consensus T helper epitopes for a cross-clade HIV vaccine. *Methods.* (2004) 34:476–87. 10.1016/j.ymeth.2004.06.003 15542374

[8] Moise L Gutierrez A Kibria F Martin R Tassone R Liu R Ivax: an integrated toolkit for the selection and optimization of antigens and the design of epitope-driven vaccines. *Hum Vaccines Immunother.* (2015) 11:2312–21. 10.1080/21645515.2015.1061159 26155959 PMC4635942

[9] Schafer JR Jesdale BM George JA Kouttab NM De Groot AS. Prediction of well-conserved HIV-1 ligands using a matrix-based algorithm. *EpiMatrix Vaccine.* (1198) 16:1880–4. 10.1016/S0264-410X(98)00173-X 9795396

[10] Bounds CE Terry FE Moise L Hannaman D Martin WD De Groot AS An immunoinformatics-derived DNA vaccine encoding human class II T cell epitopes of Ebola virus, Sudan virus, and Venezuelan equine encephalitis virus is immunogenic in HLA transgenic mice. *Hum Vaccines Immunother.* (2017) 13:2824–36. 10.1080/21645515.2017.1329788 28575582 PMC5718811

[11] Weber CA Mehta PJ Ardito M Moise L Martin B De Groot AS. T cell epitope: friend or foe? Immunogenicity of biologics in context. *Adv Drug Deliv Rev.* (2009) 61:965–76. 10.1016/j.addr.2009.07.001 19619593 PMC7103283

[12] Moise L McMurry JA Buus S Frey S Martin WD De Groot AS. In silico-accelerated identification of conserved and immunogenic variola/vaccinia T-cell epitopes. *Vaccine.* (2009) 27:6471–9. 10.1016/j.vaccine.2009.06.018 19559119 PMC2838212

[13] McMurry JA Gregory SH Moise L Rivera D Buus S De Groot AS. Diversity of *Francisella tularensis* Schu4 antigens recognized by T lymphocytes after natural infections in humans: identification of candidate epitopes for inclusion in a rationally designed tularemia vaccine. *Vaccine.* (2007) 25:3179–91. 10.1016/j.vaccine.2007.01.039 17291638

[14] Legoux FP Lim JB Cauley AW Dikiy S Ertelt J Mariani TJ CD4+ T cell tolerance to tissue-restricted self antigens is mediated by antigen-specific regulatory T cells rather than deletion. *Immunity.* (2015) 43:896–908. 10.1016/j.immuni.2015.10.011 26572061 PMC4654997

[15] Malchow S Leventhal DS Lee V Nishi S Socci ND Savage PA. Aire enforces immune tolerance by directing autoreactive T cells into the regulatory T cell lineage. *Immunity.* (2016) 44:1102–13. 10.1016/j.immuni.2016.02.009 27130899 PMC4871732

[16] Samy ET Parker LA Sharp CP Tung KSK. Continuous control of autoimmune disease by antigen-dependent polyclonal CD4+CD25+ regulatory T cells in the regional lymph node. *J Exp Med.* (2005) 202:771–81. 10.1084/jem.20041033 16172257 PMC2212949

[17] De Groot AS Moise L Liu R Gutierrez AH Tassone R Bailey-Kellogg C Immune camouflage: relevance to vaccines and human immunology. *Hum Vaccines Immunother.* (2014) 10:3570–5. 10.4161/hv.36134 25483703 PMC4514035

[18] Moise L Gutierrez AH Bailey-Kellogg C Terry F Leng Q Abdel Hady KM The two-faced T cell epitope: examining the host-microbe interface with JanusMatrix. *Human Vaccines Immunother.* (2013) 9:1577–86. 10.4161/hv.24615 23584251 PMC3974887

[19] Moise L Terry F Ardito M Tassone R Latimer H Boyle C Universal H1N1 influenza vaccine development: identification of consensus class II hemagglutinin and neuraminidase epitopes derived from strains circulating between 1980 and 2011. *Human Vaccines Immunother.* (2013) 9:1598–607. 10.4161/hv.25598 23846304

[20] Pedersen LE Harndahl M Nielsen M Patch JR Jungersen G Buus S Identification of peptides from foot-and-mouth disease virus structural proteins bound by class i swine leukocyte antigen (SLA) alleles, SLA-1^∗^0401 and SLA-2^∗^0401. *Anim Genet.* (2013) 44:251–8. 10.1111/j.1365-2052.2012.02400.x 22984928

[21] Lamont EA Poulin E Sreevatsan S Cheeran MCJ. Major histocompatibility complex i of swine respiratory cells presents conserved regions of influenza proteins. *J Gen Virol.* (2018) 99:303–8. 10.1099/jgv.0.001008 29458525

[22] Falk K Rötzschke O Stevanovié S Jung G Rammensee HG. Allele-specific motifs revealed by sequencing of self-peptides eluted from MHC molecules. *Nature.* (1991) 351:290–6. 10.1038/351290a0 1709722

[23] Tungatt K Dolton G Morgan SB Attaf M Fuller A Whalley T Induction of influenza-specific local CD8 T-cells in the respiratory tract after aerosol delivery of vaccine antigen or virus in the Babraham inbred pig. *PLoS Pathog.* (2018) 14:e1007017. 10.1371/journal.ppat.1007017 29772011 PMC5957346

[24] Pedersen LE. *Analysis of Swine Leukocyte Antigen Peptide Binding Profiles and the Identification of T cell Epitopes by Tetramer Staining.* (2012). Ph.D. thesis, Technical University of Denmark, Lyngby.

[25] Sturniolo T Bono E Ding J Raddrizzani L Tuereci O Sahin U Generation of tissue-specific and promiscuous HLA ligand databases using DNA microarrays and virtual HLA class II matrices. *Nat Biotechnol.* (1999) 17:555–61. 10.1038/9858 10385319

[26] Stern LJ Brown JH Jardetzky TS Gorga JC Urban RG Strominger JL Crystal structure of the human class II MHC protein HLA-DR1 complexed with an influenza virus peptide. *Nature.* (1994) 368:215–21. 10.1038/368215a0 8145819

[27] Sinigaglia F Hammer J. Defining rules for the peptide-MHC class II interaction. *Curr Opin Immunol.* (1994) 6:52–6. 10.1016/0952-7915(94)90033-7 7513526

[28] Zhang L Chen Y Wong HS Zhou S Mamitsuka H Zhu S. Tepitopepan: extending tepitope for peptide binding prediction covering over 700 HLA-DR molecules. *PLoS One.* (2012) 7:e30483. 10.1371/journal.pone.0030483 22383964 PMC3285624

[29] Zhang H Lund O Nielsen M. The pickpocket method for predicting binding specificities for receptors based on receptor pocket similarities: application to MHC-peptide binding. *Bioinformatics.* (2009) 25:1293–9. 10.1093/bioinformatics/btp137 19297351 PMC2732311

[30] Baratelli M Pedersen LE Trebbien R Larsen LE Jungersen G Blanco E Identification of cross-reacting T-cell epitopes in structural and non-structural proteins of swine and pandemic H1N1 influenza a virus strains in pigs. *J Gen Virol.* (2017) 98:895–9. 10.1099/jgv.0.000748 28555545 PMC5563543

[31] Gutiérrez AH Loving C Moise L Terry FE Brockmeier SL Hughes HR In vivo validation of predicted and conserved T cell epitopes in a swine influenza model. *PLoS One.* (2016) 11:e0159237. 10.1371/journal.pone.0159237 27411061 PMC4943726

[32] Southwood S Sidney J Kondo A del Guercio MF Appella E Hoffman S Several common HLA-DR types share largely overlapping peptide binding repertoires. *J Immunol.* (1998) 160:3363–73. 9531296

[33] Sidney J Peters B Frahm N Brander C Sette A. HLA class I supertypes: a revised and updated classification. *BMC Immunol.* (2008) 9:1. 10.1186/1471-2172-9-1 18211710 PMC2245908

[34] Pedersen LE Jungersen G Sorensen MR Ho CS Vadekær DF. Swine leukocyte antigen (SLA) class I allele typing of Danish swine herds and identification of commonly occurring haplotypes using sequence specific low and high resolution primers. *Vet Immunol Immunopathol.* (2014) 162:108–16. 10.1016/j.vetimm.2014.10.007 25457547

[35] Ho CS Lunney JK Franzo-Romain MH Martens GW Lee YJ Lee JH Molecular characterization of swine leucocyte antigen class i genes in outbred pig populations. *Anim Genet.* (2009) 40:468–78. 10.1111/j.1365-2052.2009.01860.x 19392823

[36] Ho CS Lunney JK Lee JH Franzo-Romain MH Martens GW Rowland RRR Molecular characterization of swine leucocyte antigen class II genes in outbred pig populations. *Anim Genet.* (2010) 41:428–32. 10.1111/j.1365-2052.2010.02019.x 20121817

[37] Hammer SE Ho C-S Ando A Rogel-Gaillard C Charles M Tector M Importance of the major histocompatibility complex (Swine Leukocyte Antigen) in Swine health and biomedical research. *Annu Rev Anim Biosci.* (2020) 8:171–98. 10.1146/annurev-animal-020518-115014 31846353

[38] Boegel S Löwer M Schäfer M Bukur T de Graaf J Boisguérin V HLA typing from RNA-Seq sequence reads. *Genome Med.* (2012) 4:102. 10.1186/gm403 23259685 PMC4064318

[39] Erlich RL Jia X Anderson S Banks E Gao X Carrington M Next-generation sequencing for HLA typing of class I loci. *BMC Genomics.* (2011) 12:42. 10.1186/1471-2164-12-42 21244689 PMC3033818

[40] Kita YF Ando A Tanaka K Suzuki S Ozaki Y Uenishi H Application of high-resolution, massively parallel pyrosequencing for estimation of haplotypes and gene expression levels of swine leukocyte antigen (SLA) class i genes. *Immunogenetics.* (2012) 64:187–99. 10.1007/s00251-011-0572-2 21932051

[41] Sørensen MR Ilsøe M Strube ML Bishop R Erbs G Hartmann SB Sequence-based genotyping of expressed swine leukocyte antigen class I alleles by next-generation sequencing reveal novel swine leukocyte antigen class I haplotypes and alleles in Belgian, Danish, and Kenyan fattening pigs and Göttingen minipigs. *Front Immunol.* (2017) 8:701. 10.3389/fimmu.2017.00701 28670315 PMC5472656

[42] Gutiérrez AH Rapp-Gabrielson VJ Terry FE Loving CL Moise L Martin WD T-cell epitope content comparison (EpiCC) of swine H1 influenza A virus hemagglutinin. *Influenza Other Respi Viruses.* (2017) 11:531–42. 10.1111/irv.12513 29054116 PMC5705686

[43] Bandrick M Gutiérrez AH Desai P Rincon G Martin WD Terry FE T cell epitope content comparison (EpiCC) analysis demonstrates a bivalent PCV2 vaccine has greater T cell epitope overlap with field strains than monovalent PCV2 vaccines. *Vet Immunol Immunopathol.* (2020) 223:110034. 10.1016/j.vetimm.2020.110034 32278900

[44] Wideman G. Impact of influenza A in pork production. *Proceedings of the London Swine Conference*, Stratford, ON (2013). p. 191–3.

[45] Sandbulte MR Spickler AR Zaabel PK Roth JA. Optimal use of vaccines for control of influenza a virus in swine. *Vaccines.* (2015) 3:22–73. 10.3390/vaccines3010022 26344946 PMC4494241

[46] Vincent AL Ciacci-Zanella JR Lorusso A Gauger PC Zanella EL Kehrli ME Efficacy of inactivated swine influenza virus vaccines against the 2009 A/H1N1 influenza virus in pigs. *Vaccine.* (2010) 28:2782–7. 10.1016/j.vaccine.2010.01.049 20132919

[47] Brandenburg B Koudstaal W Goudsmit J Klaren V Tang C Bujny MV Mechanisms of hemagglutinin targeted influenza virus neutralization. *PLoS One.* (2013) 8:e80034. 10.1371/journal.pone.0080034 24348996 PMC3862845

[48] La Gruta NL Turner SJ. T cell mediated immunity to influenza: mechanisms of viral control. *Trends Immunol.* (2014) 35:396–402. 10.1016/j.it.2014.06.004 25043801

[49] Altenburg AF Rimmelzwaan GF de Vries RD. Virus-specific T cells as correlate of (cross-)protective immunity against influenza. *Vaccine.* (2015) 33:500–6. 10.1016/j.vaccine.2014.11.054 25498210

[50] Mcmichael AJ Gotch FM Noble GR Beare PAS. Cytotoxic T-cell immunity to influenza. *N Engl J Med.* (1983) 309:13–7. 10.1056/NEJM198307073090103 6602294

[51] Hewitt JS Karuppannan AK Tan S Gauger P Halbur PG Gerber PF A prime-boost concept using a T-cell epitope-driven DNA vaccine followed by a whole virus vaccine effectively protected pigs in the pandemic H1N1 pig challenge model. *Vaccine.* (2019) 37:4302–9. 10.1016/j.vaccine.2019.06.044 31248687

[52] Sánchez-Vizcaíno JM Mur L Martínez-López B. African Swine fever: an epidemiological update. *Transbound Emerg Dis.* (2012) 59 (Suppl. 1):27–35. 10.1111/j.1865-1682.2011.01293.x 22225967

[53] Sánchez-Vizcaíno JM Mur L Gomez-Villamandos JC Carrasco L. An update on the epidemiology and pathology of African swine fever. *J Comp Pathol.* (2015) 152:9–21. 10.1016/j.jcpa.2014.09.003 25443146

[54] Penrith ML Vosloo W. Review of African swine fever: transmission, spread and control. *J S Afr Vet Assoc.* (2009) 80:58–62. 10.4102/jsava.v80i2.172 19831264

[55] Galindo I Alonso C. African swine fever virus: a review. *Viruses.* (2017) 9:103. 10.3390/v9050103 28489063 PMC5454416

[56] Barasona JA Gallardo C Cadenas-Fernández E Jurado C Rivera B Rodríguez-Bertos A First oral vaccination of eurasian wild boar against African Swine fever virus genotype II. *Front Vet Sci.* (2019) 6:137. 10.3389/fvets.2019.00137 31106218 PMC6498142

[57] Costard S Wieland B De Glanville W Jori F Rowlands R Vosloo W African swine fever: how can global spread be prevented? *Philos Trans R Soc B Biol Sci.* (2009) 364:2683–96. 10.1098/rstb.2009.0098 19687038 PMC2865084

[58] Gallardo MC Reoyo A de la T Fernández-Pinero J Iglesias I Muñoz MJ Arias ML. African swine fever: a global view of the current challenge. *Porc Heal Manag.* (2015) 1:21. 10.1186/s40813-015-0013-y 28405426 PMC5382474

[59] Dixon LK Abrams CC Bowick G Goatley LC Kay-Jackson PC Chapman D African swine fever virus proteins involved in evading host defence systems. *Vet Immunol Immunopathol.* (2004) 100:117–34. 10.1016/j.vetimm.2004.04.002 15207450

[60] Reis AL Netherton C Dixon LK. Unraveling the armor of a killer: evasion of host defenses by African swine fever virus. *J Virol.* (2017) 80:10487–96. 10.1128/jvi.02338-16 28031363 PMC5331812

[61] King K Chapman D Argilaguet JM Fishbourne E Hutet E Cariolet R Protection of European domestic pigs from virulent African isolates of African swine fever virus by experimental immunisation. *Vaccine.* (2011) 29:4593–600. 10.1016/j.vaccine.2011.04.052 21549789 PMC3120964

[62] Monteagudo PL Lacasta A López E Bosch L Collado J Pina-Pedrero S BA71ΔCD2: a new recombinant live attenuated african swine fever virus with cross-protective capabilities. *J Virol.* (2017) 91:e01058-17. 10.1128/jvi.01058-17 28814514 PMC5640839

[63] Burmakina G Malogolovkin A Tulman ER Zsak L Delhon G Diel DG African swine fever virus serotype-specific proteins are significant protective antigens for African swine fever. *J Gen Virol.* (2016) 97:1670–5. 10.1099/jgv.0.000490 27114233

[64] Oura CAL Denyer MS Takamatsu H Parkhouse RME. In vivo depletion of CD8+ T lymphocytes abrogates protective immunity to African swine fever virus. *J Gen Virol.* (2005) 86(Pt 9):2445–50. 10.1099/vir.0.81038-0 16099902

[65] Escribano JM Galindo I Alonso C. Antibody-mediated neutralization of African swine fever virus: myths and facts. *Virus Res.* (2013) 173:101–9. 10.1016/j.virusres.2012.10.012 23159730

[66] Rock DL. Challenges for African swine fever vaccine development—“…perhaps the end of the beginning.”. *Vet Microbiol.* (2017) 206:52–8. 10.1016/j.vetmic.2016.10.003 27756505

[67] Goatley LC Reis AL Portugal R Goldswain H Shimmon GL Hargreaves Z A pool of eight virally vectored African swine fever antigens protect pigs against fatal disease. *Vaccines.* (2020) 8:234. 10.3390/vaccines8020234 32443536 PMC7349991

[68] Kitikoon P Nilubol D Erickson BJ Janke BH Hoover TC Sornsen SA The immune response and maternal antibody interference to a heterologous H1N1 swine influenza virus infection following vaccination. *Vet Immunol Immunopathol.* (2006) 112:117–28. 10.1016/j.vetimm.2006.02.008 16621020

[69] Gramer M Rossow K. Epidemiology of swine influenza and implications of reassortment. In: Allen D editor. *Proceedings of the Leman Swine Conference.* Minneapolis, MN: University of Minnesota Digital Conservancy (2004). p. 69–73.

[70] Rapp-Gabrielson V Lenz MC Hildebrand T Taylor L Kuhn M. Evaluation of cross-protection of FluSure XP^®^ against a heterologous gamma cluster H1N1 swine influenza virus challenge. In: Allen D editor. *Proceedings of the Leman Swine Conference.* (Vol. 267), Minneapolis, MN: University of Minnesota Digital Conservancy. (2011).

[71] Rapp-Gabrielson V Lenz M Kuhn M Taylor LP Culhane M Kesl L Cross-protection of FluSure XP(R) in pigs challenged with a gamma cluster H1N1/pH1N1 reassortant swine influenza virus. *Proceedings of the AASV Annual Meet.* (2014). Available online at: https://www.aasv.org/library/swineinfo/

[72] Detmer SE Gramer MR King VL Mathur S Rapp-Gabrielson VJ. In vivo evaluation of vaccine efficacy against challenge with a contemporary field isolate from the α cluster of H1N1 swine influenza virus. *Can J Vet Res.* (2013) 77:24–32. 23814353 PMC3525169

[73] Chattha KS Roth JA Saif LJ. Strategies for design and application of enteric viral vaccines. *Annu Rev Anim Biosci.* (2015) 3:375–95. 10.1146/annurev-animal-022114-111038 25387111

[74] Zimmerman JJ Karriker LA Ramirez A Schwartz KJ Stevenson GW. *Disease of Swine.* 10th ed Ames, IA: Wiley-Blackwell (2012). 10.1017/CBO9781107415324.004

[75] Vlasova AN Amimo JO Saif LJ. Porcine rotaviruses: epidemiology, immune responses and control strategies. *Viruses.* (2017) 9:48. 10.3390/v9030048 28335454 PMC5371803

[76] Papp H László B Jakab F Ganesh B De Grazia S Matthijnssens J Review of group A rotavirus strains reported in swine and cattle. *Vet Microbiol.* (2013) 165:190–9. 10.1016/j.vetmic.2013.03.020 23642647 PMC7117210

[77] Khan S Tan S Shepherd F Gutierrez A Moise L Marthaler D Application of the Epitope Content Comparison Tool (EpiCC) to develop better swine rotavirus vaccines. *Procedeings of the 99th Conference of Research Workers in Animal Diseases.* Chicago, IL. (2018).

[78] Franzo G Segalés J. Porcine circovirus 2 (PCV-2) genotype update and proposal of a new genotyping methodology. *PLoS One.* (2018) 13:e0208585. 10.1371/journal.pone.0208585 30521609 PMC6283538

[79] Karuppannan AK Opriessnig T. Porcine circovirus type 2 (PCV2) vaccines in the context of current molecular epidemiology. *Viruses.* (2017) 9:99. 10.3390/v9050099 28481275 PMC5454412

[80] Welner S Nielsen M Rasmussen M Buus S Jungersen G Larsen LE. Prediction and in vitro verification of potential CTL epitopes conserved among PRRSV-2 strains. *Immunogenetics.* (2017) 69:689–702. 10.1007/s00251-017-1004-8 28589207 PMC5597684

[81] Gao C He X Quan J Jiang Q Lin H Chen H Specificity characterization of SLA class I molecules binding to swine-origin viral cytotoxic T lymphocyte epitope peptides in vitro. *Front Microbiol.* (2017) 8:2524. 10.3389/fmicb.2017.02524 29326671 PMC5741678

[82] Van Chanh Le Q Le TM Cho HS Kim W Il Hong K Song H Analysis of peptide-SLA binding by establishing immortalized porcine alveolar macrophage cells with different SLA class II haplotypes. *Vet Res.* (2018) 49:96. 10.1186/s13567-018-0590-2 30241566 PMC6151021

[83] Lyamichev VI Goodrich LE Sullivan EH Bannen RM Benz J Albert TJ Stepwise evolution improves identification of diverse peptides binding to a protein target. *Sci Rep.* (2017) 7:12116. 10.1038/s41598-017-12440-1 28935886 PMC5608804

[84] Parrillo M Gutierrez A Moise L Dakouo M Traoré A Sacko S “On demand” vaccine design: application to African Swine fever virus. *Procedeings of the 98th Conference of Research Workers in Animal Diseases.* Chicago, IL. (2017).

